# Efficacy of 400 mg albendazole against soil‐transmitted helminthes among Salgy Primary School Children, Dembia district, Northwest Ethiopia, 2020. “Uncontrolled experimental study”

**DOI:** 10.1002/hsr2.2041

**Published:** 2024-04-17

**Authors:** Adane Assefa, Demekech Damtie, Getu Abeje, Andargachew Almaw, Ayenew Berhan, Habtu Debsh, Molla Getie

**Affiliations:** ^1^ Department of Medical Parasitology, College of Medical and Health Sciences University of Gondar Gondar Ethiopia; ^2^ Department of Biomedical sciences, College of Medical and Health Sciences Samara University Samara Ethiopia; ^3^ Department of Medical Laboratory Sciences, College of Medical and Health Sciences Debre Tabor University Debre Tabor Ethiopia; ^4^ Department of Medical Laboratory Sciences, College of Medical and Health Sciences Wollo University Dessie Ethiopia; ^5^ Department of Medical Laboratory Sciences, College of Medical and Health Sciences Inji‐bara University inje‐bara Ethiopia

**Keywords:** albendazole, cure rate, egg reduction rate, Ethiopia, soil‐transmitted helminths

## Abstract

**Background and Aims:**

Soil‐transmitted helminths are one of the most prevalent causes of both intellectual and physical disability in the world. Albendazole (ALB) is a drug recommended for mass treatment of the high burden of soil‐transmitted helminths in schoolchildren, particularly in developing countries. However, some researchers have reported that the efficacy of albedazole against soil‐transmitted helminths is inconsistent. Monitoring the programs is crucial to evaluating the effectiveness of 400 mg of ALB against soil‐transmitted helminths, as well as any changes in its therapeutic efficacy. Thus, the purpose of this study was to evaluate ALB effectiveness in treating soil‐transmitted helminthes in Salgy Primary School Children.

**Methods:**

An uncontrolled experimental study was conducted at Salgy Primary School Children, Northwest Ethiopia, from March to May 2020. A total of 439 schoolchildren were enrolled and screened for soil‐transmitted helminths by stratified proportionate systematic random sampling to get 228 positive schoolchildren. Students in grades one through eight were grouped based on their educational attainment. Using the Kato‐Katz thick smear technique, the selected stool sample collected from school children was examined using the Kato‐Katz thick smear technique to determine the cure and egg reduction rates. The statistical package for social science software, version 20, was used to analyze the data. To determine the relationship between CR (cure rate) and ERR (egg reduction rate) by age, a chi‐square test (*X* ^2^) was employed and significance was considered at A 95% confidence interval and *p* Value (*p* < 0.05).

**Results:**

A 400 mg single dosage of ALB showed a 99.35% CR and a 97.30% egg reduction rate against *Ascaris lumibricoides*. Additionally, a 400 mg dose of ALB showed a 95.75% CR and an 82.07% egg reduction rate, suggesting questionable effectiveness against hookworm infections. *Trichuris trichiura* showed a decreased efficacy, with a 43.53% CR and a 23.12% egg reduction rate.

**Conclusion:**

A single dose of 400 mg ALB is effective (satisfactory), doubtful, and unsatisfactory against *Ascaris lumbricoides*, hookworm, and *T. trichiura* infections, respectively. Further studies using different brands, doses, and routes will be needed to treat hookworm and *T. trichiura* infections successfully by using a larger sample size.

## 1 INTRODUCTION

Intestinal parasite infections represent a significant public health issue in the world. Globally, 3.5 billion and 450 million people are infected and ill, respectively, while the majority of them are children,^1^ particularly in developing countries.^2^ Soil‐transmitted helminthiasis (STH) are widely distributed, and continues to be a serious public health issue in the poorest areas.^3^ The majority of them are caused by three human helminth worms. Such as the roundworm (*Ascaris lumbricoides*), the whipworm (*Trichuris trichiura*), and two hookworm species (*Ancylostoma duodenale* and *Necator americanus*).^4^ This worm can spread among people by eating contaminated food, water, or soil containing eggs, and the later by active penetrating of the larvae through skin in the soil.^5^ All age groups are impacted, but school‐age children are most affected worldwide, there are over 400 million school‐age children affected which can cause malnourishment, stunted growth, intellectual retardation, and problems with cognition and learning ability.^6^, ^7^, ^8^ It is the second‐leading cause of mortality in Africa, particularly under‐5‐year children.^9^

Therefore, frequent antihelminthic therapy, better water supply (sanitation), and health education^10^ can serve as the foundation for managing STHs. The Public health initiatives aimed for reducing the morbidity associated to STHs primarily depend on giving anti‐helminthic medications to elementary school students.^11^ Albendazole (ALB) is a derivative of the benzimidazole drug, which is one of the most widely used drugs to treat STH infections.^8^, ^12^ It is believed to work by inhibiting tubulin polymerization^13^ and has been used extensively in human clinical medicine as a safe anti‐helminthic agent that exhibits larvicidal, ovicidal, and vermicidal activity. Due to a series of metabolic disruptions including energy depletion, the sensitive helminths become immobile and eventually die.^13^

To reduce morbidity associated with STH infections, periodic administration of ALB to high and moderate risk populations is the key strategy worldwide.^11^ A single 400 mg ALB dose has been shown to be quite effective against *A. lumbricoides* and hookworm infections, but ineffective against *T. trichiura* infections.^14^, ^15^ Furthermore, the effectiveness of ALB for treatment of *T. trichiura* can differ depending on the severity of the infection. ALB exhibits high efficacy in low‐infection of *T. trichiura* cases and poor efficacy in high‐infection cases.^16^ However, combination of 400 mg of ALB and 500 mg of mebendazole as single dose has a better cure rate (CR) and reduces the number of eggs in *T. trichiura* infections than 400 mg ALB alone.^17^

The efficacy of anti‐helminthic drugs in humans can be confounded by different methodologically variations, like treatment regimens, poor quality of drugs, and statistical analysis for calculating therapeutic efficacy methods such as egg reduction rates. In addition, other factors, like study design, small sample size, variation in pre‐intervention intensities, type of diagnostic tests, and geographical location, may confound the efficacy of anti‐helminthic drugs.^18^ A scale‐up of periodic delivery of chemotherapy programs to schoolchildren can increase drug pressure on parasite populations and may favor parasite genotypes to be resistant to common anti‐helminthic drugs.^19^ Knowing the occurrence and trends of anti‐helminthic resistance is important to overcome, slow the spread, delay the development of resistance to new anti‐helminthic drugs, as well as for better management of parasite control^20^ including the use of anti‐helminthic combinations with the existing anthelminthic drugs.^20^

More‐ever, single doses of ALB in school‐age children who harbor a heavy intestinal worm burden has significantly reduced infection.^21^, ^22^, ^23^, ^24^ Monitoring drug efficacy and the assessment can increase detection of the emerging resistance of drugs.^25^ The drugs has been frequently used in different parts of the world. Regular monitoring of the drug's effectiveness is crucial for ensuring therapeutic reliability. CR and ERR are the two most commonly utilized indicators to evaluate the effectiveness of anti‐helminthic medications. That being said, ERR is more significant in controlling STH than CR, even though CR is regarded as a crucial efficacy indicator.^25^

Accordingly a few earlier studies found that, for some STH (*A. lumbercoides* and H. worm), ERR and CR for a single dose of ALB are more effective, whereas for other STH (*T. trichuris*), they are less effective in some part of Ethiopia. However, some other studies showed that, there is some difference in efficacy of ALB against STH. Therefore, more information from various situations is needed to resolve the disparity and influence policymakers so that more research will be recommended for assessing the efficacy of anti‐helminthic drugs. As a result, the current efficacy research, carried out at Salgy Primary School in the Dembia district Northwest Ethiopia, used to determine CR and ERR of a 400 mg single dosage of ALB (manufactured by India Ipca Laboratory Ltd.) against STH infections. Then, the study will serve as baseline information for future evaluation of ALB interventions against STH infections in the area, region, and country.

## 2 MATERIALS AND METHODS

### 2.1 Study area

This study was conducted from Salgy Primary School Children in the Dembia District. It is situated 780 km from Addis Ababa, Ethiopia. It is found within an altitude range between 1750 and 2100 m above sea level. The district has 101 primary schools and 5 high schools, including one preparatory school. A total of 1364 schoolchildren (655 male and 709 female) are found in Salgy Primary School. In this district, almost 92% of the total population has been get an access to visit by health extension workers. The district lies on the shore of Lake Tana and has a total population of more than 300,000 and majority them depend on subsistence farming (source: *Woreda* office of agriculture).

### 2.2 Study design and period

An uncontrolled experimental study was conducted from March to May 2020 at Salgy Primary School Children, Dembia District, NorthWest Ethiopia.

### 2.3 Eligibility criteria

Primary school children whose age is between 6 and 15 years and who have lived in the study area for at least 1 month and whose parents or guardians were willing to participate in the study were included. Schoolchildren who had diarrhea and vomiting within 4 h after drug administration, a recent history of anthelminthic drug treatment (ALB, mebendazole, pyrantel, and levamisole) within the past 4 weeks, known or reported hypersensitivity to ALB, and children with chronic illness or acutely ill children (any disease) whose guardian was unwilling to give written consent were excluded.

### 2.4 Sample size and sampling technique

Using the single population proportion formula the total sample size was 228 by using the predicted efficacy of ALB for *A. lumbricoides* and^26^ hook worms of 83.9% with a 10% nonresponse rate.

$$n=Z^{2}E\;\left(1-E\right)/d^{2}$$

Where n is the sample size. *Z* = *Z* statistic at 95% confidence interval (CI).

*d* refers to precision, and *E* = estimated effectiveness of the ALB therapy.

n=1.96*0.839(1−0.839)=207

For *A. lumbricoides* and hookworm the estimated efficacy of ALB was 83.9%.

Thus, the total sample size after calculating the sample size and adding a 10% nonresponse rate was 228. Lastly, the students were chosen using a stratified proportionate systematic random sampling technique. The total number of students from grades one through eight was determined. The students were categorized based on their educational level from grades one to eight. The students were assigned to grade levels based on the proportion of students in each grade. Lastly, the students were chosen using a systematic random sampling technique using the class roster as the sampling frame.

### 2.5 Operational definition

CR: The proportion of treated children who have no longer eggs.

$$\mathrm{CR}=\frac{\left(\text{children}\;\text{negative}\;\text{three}\;\text{weeks}\;\text{post}‐\text{treatment}\right)}{\mathrm{Children}\;\mathrm{positive}\;\mathrm{for}\;\mathrm{parasitic}\;\mathrm{infection}\;\mathrm{pre}‐\mathrm{treatment}}\times 100\%$$

Egg Reduction Rate (ERR): A decrease from baseline in the mean fecal egg count at treatment follow‐up

$$\text{ERR}\;\text{is}\;\text{equal}\;\text{to}\;\frac{\left(1-\text{arithmetic}\;\text{mean}\;\text{of}\;\text{egg}/\text{gram}\;\text{after}\;\text{treatment}\right)}{\mathrm{Arithmetic}\;\mathrm{mean}\;\mathrm{of}\;\mathrm{egg}/\mathrm{gram}\;\mathrm{previously}\;\mathrm{treated}}\times 100\%,$$

The pharmacological efficacy of ALB (400 mg chewable tablet) is calculated differently in Brazil, Cambodia, Cameroon, Ethiopia, India, the United Republic of Tanzania, and Vietnam.^27^ This was described by three most prevalence STH. Such as *A. lumbricoides*, Hookworm and *T. trichiura* with their reference efficacy (egg reduction rate, %)* ≥95, ≥90 and ≥50*, respectively. The ERR in cases of *T. trichiura* infection is much lower than that for the other STHs, according to a study that involved 1834 people from Brazil, Cambodia, Cameroon, Ethiopia, India, the United Republic of Tanzania, and Viet Nam. In addition, there are guidelines and class of EPG (Egg per Gram) from the intensity of Soil‐transmitted helminth infections at community level^28^ from the three STH like; *A. lumbricoides, T. trichiura and* Hookworm have 1−4999, 5000−49,999 and ≥50,000; 1−999, 1000−9999 and ≥10,000; 1−1999, 2000−3999 and ≥4000 light intensity infections, moderate intensity infections and heavy intensity Infections, respectively.

Note: “*” refers strikes which important to describe similar points to avoid repetition.

The efficacy of anthelminthic drugs is^27^

- Sattisfactory; if the ERR is higher than or equal to the reference value.
- Reduced: if the ERR is below the reference value by at least 10% points
- Doubtful: if the ERR is below the reference value by less than 10% points.

#### 2.5.1 Definition statistical terms

*X* ^2^ = The Chi‐square test is a nonparametric statistical test used to determine if there's a significant association between two or more categorical variables in a sample. *p*, or probability, expresses the likelihood that any difference between groups that is seen is the result of chance. It has any value between 0 and 1. *p* values near 1 suggest no difference between the groups other than chance, while values near 0 indicate that the observed difference is unlikely to be the result of chance. Because of this, it is typical for medical publications to follow a P value with an adjective like “very significant” or “highly significant.

### 2.6 Data collection and laboratory methods

#### 2.6.1 Sample collection and processing

Schoolchildren were given explicit instructions before giving a feces sample using a dry, clean, and leak‐proof plastic stool cup. Two Kato‐Katz smears were prepared for each specimen using a standardized template containing 41.7 mg of stool sample. Upon receipt, one slide was promptly refrigerated (at about 5°C), while the other was kept at ambient temperature (about 25°C), then they were examine under a microscope to determine the number of STH eggs.

After 20 min, the first microscopically analysis was performed on both Kato‐Katz slides. An hour after stool collection and Kato slide preparation, the hookworm egg count and intensity was carried out. The intensity was calculated by multiplying the egg count per slide by 24. Only schoolchildren who were positive for any of the three soil‐transmitted helminths were used as the baseline to be involved in the intervention and provided a single dose of 400 mg ALB through swallowing. These were followed‐up for 21 days and provided a second stool sample to determine ERR and CR. A single dose of 400 mg ALB was also provided again for those school children who were positive for the post treatment. Health education was given by health professionals to study participants who were positive for any of the three STHs provided for swallowing a single dose of 400 mg ALB.

#### 2.6.2 Drug administration and follow‐up

A single standard dose (400 mg) of ALB (Manufactured by India, Ipca Laboratories Ltd.) from the same manufacturer and similar batch number was administered to STH positive school children. The tablet was administered under direct observation (nurses), and the schoolchildren were maintained under observation for approximately 4 h.

### 2.7 Quality control

A standard amount of specimen was used to prepare the Kato‐Katz procedure, and examined in a predetermined amount of time. To validate the reports, the egg counts differed by more than 10% of the slide were randomly selected re‐examined at the end by two senior experienced laboratory technicians who were blind to the results of the initial examination. For any discrepancies (positive vs negative results and egg counts differing by >10%), results were discussed with the respective technicians and decided together. Finally, the results of laboratory examination were recorded in well‐prepared format carefully.

### 2.8 Data analysis and interpretation

The Statistical Package for Social Science Software version 20 was used to code, enter, clean, and analyze the data. Chi‐square test (*X* ^2^) was used to manipulate the data to explain the association between CR and ERR by age. The reduction of infected children and fecal egg counts was used to assess the effectiveness of the treatment for each STH, and the mean of the pre‐ and postintervention fecal egg counts was used to calculate the fecal egg count reduction rate. Furthermore, the percentage of individuals who became parasitological negative after treatment was used to calculate the CR. The level of egg excretion intensity was determined at the pre‐ and postintervention survey. The corresponding two‐sided 95% CI had been used to show the association's strength. Significance was considered at 95% CI and *p* Value (*p* < 0.05).

### 2.9 Ethics approval and consent to participate

Before the study started, ethical approval (project code: 100/2020) was received from the University of Gonder College of Medicine and Health Sciences Ethical Review Committee. APHI provided a letter of authorization, and other letters of support were acquired from Salgy Primary School, the educational bureau, the Dembia district health office, and the North Gonder Zonal Department. Study participants and their parents/guardians were informed about the study's purpose. The child's parents or legal guardians provided written, informed consent. The data was kept private and secret. Children with soil‐transmitted helminth infections were treated in accordance with national protocols, as well as standards outlined in the Declaration of Helsinki.

## 3 RESULTS

### 3.1 Characteristics of the population

The sum of 439 schoolchildren were screened for STH by the Kato‐Katz technique, and 228 schoolchildren were enrolled in the study.

#### 3.1.1 Pre‐ and post‐treatment prevalence of helminth infections

Of the 439 schoolchildren participated in the study; 228 (52%) of them were infected with one or more helminth parasites. The prevalence of hookworm, *A. lumbricoides*, and *T. trichuria* infections was only 50 (22%), 45 (19.7%), and 9 (4%), respectively. In addition, double‐ and triple‐mixed infections of STH occurred. The double infections of *Ascaris‐Trichuris*, *Ascaris*‐hookworm, and *Trichuris*‐hookworm were 57 (25%), 48 (21%), and 9 (4%), respectively, and the triple mixed infection of Ascaris‐Trichuris‐hookworm was 10 (4.4%) was observed. In the post‐treatment period, the positivity percentage rates of *A. lumbricoides* and hookworm infection showed 1.9% (1) and 9.4% (5), respectively, but the percentage positivity rate for *T. trichiura* infection was increased to 90.6% (Table 1).

**Table 1:** Efficacy of albendazole against *Ascaris lumbricoides*, *Trichuris trichiura*, and Hookworm in 3 weeks post treatment among school children in Salgy Primary School, Dembia District, and Northwest Ethiopia from March to May, 2020.

STH	% (Number infected)		CR (%)	Arithmetic mean(epg)		ERR (%)
				Pretreatment	Post‐treatment	
	Pretreatment	Post‐treatment				
Ascariasis	70.2 (160)	1.9 (1)	99.3	3561.82	96.00	97.30
Hookworm Infection	51.3 (117)	9.4 (5)	95.75	187.37	33.60	82.07
Trichuriasis	37.3 (85)	90.6 (48)	43.53	77.40	59.50	23.12

Abbreviations: CR, cure rate; epg, egg per gram.

#### 3.1.2 CR and egg reduction rate

For *A. lumbricoides*, a single 400 mg dose of ALB had a CR of 99.3% and an ERR of 97.3%, and against hookworm infection, it had a CR of 95.75% and an ERR of 82.12%, respectively, while the CR and ERR against *T. trichiura* were 43.53% and 23.12%, respectively. Following the initial 3‐week treatment, over 76.3% of STH‐infected children recovered, and the overall egg production dropped by over 80.3%. Before treatment the proportions of moderate and light infections for *A. lumbricoides*, *T. trichiura* and Hookworm were; 20.2% (46/228) and 50.0% (114/228), 0.4% (1/228) and 36.8% (84/228), 0.4% (1/228), and 50.9% (116/228), respectively. Heavy infection was not observed in all the three STH. In the post treatment, moderate and light infections were observed for *T. trichuris*, *A. lumbricoides* and hookworm infections which showed a good reduction potential. Heavy infection was not observed in all three STHs (Table 2).

**Table 2:** Pre‐ and post‐treatment intensity of infection for *Ascaris lumbricoides*, *Trichuris trichiura*, and hookworm species among school children in Salgy Primary School, Dembia District, Northwest Ethiopia, from March to May 2020.

Infection Intensity	Percent (Number) of infected					
	*A. lumbricoides*		Hookworm		*T. trichiura*	
	Pretreatment	Post‐treatment	Pretreatment	Post‐treatment	Pretreatment	Post‐treatment
Heavy	0 (0%)	0 (0%)	0 (0%)	0 (0%)	0 (0%)	0 (0%)
Moderate	46 (20.2%)	0 (0%)	1 (0.4%)	0 (0%)	1 (0.4%)	1 (1.9%)
Light	114 (50.0%)	1 (1.9%)	116 (50.9%)	5 (9.4%)	84 (6.8%)	47 (88.7%)

*Note*: Association of age and sex with cure rate and egg reduction rate.

#### 3.1.3 Association between age and ALB cure and egg decrease rates

The CR of A. lumbricoides, and hookworm infections with age did not appear to be significantly associated (*p* = 0.69 and 0.33, respectively, or *p* > 0.05). While, a significant association between age and CR of *T. trichiura* infection was observed (*p* = 0.02, *p* < 0.04), but an insignificant association was observed in the ERR of the three STHs. Regarding age, in hookworm and *A. lumbercoides*, the infection increases with age, yet in trichuriasis, the percentage' of infection decreases with age (Tables 3 and 4).

**Table 3:** Cure rate of albendazole by age at Salgy Primary School Children, Dembia District, Northwest, Ethiopia from March to May, 2020.

STH Infection	Number (%) of infected							
	Pretreatment			Post‐treatment			*p* Value	*X* ^2^
	6−10 years	11−15 years	Total	6−10 years	11−15 years	Total		
Ascariasis	58 (36.2)	102 (63.8)	160 (100)	0 (0)	1 (0.63)	1 (0.63)	0.69	0.76
*Hookworm* Infection	18 (15.4)	99 (84.6)	117 (100)	1 (0.85)	4 (3.42)	5 (4.27)	0.33	0.95
Trichuriasis	62 (73)	23 (27)	85 (100)	32 (37.6)	16 (13.7)	48 (56.5)	0.02	1.51

Abbreviation: *X* ^2^, Chi‐square.

**Table 4:** Egg reduction rate of albendazole by age of Salgy Primary School, Dembia District, Northwest Ethiopia from March to May, 2020.

STH Infection	Number (%) of infected							
	Pretreatment *x* ^2^			Post‐treatment			*p* Value	*X* ^2^
	6−10 years	11−15 years	Total	6−10 years	11−15 years	Total		
Ascariasis	58 (36.2)	102 (63.8)	160 (100)	0 (0)	1 (0.63)	1 (0.63)	0.76	0.14
*Hookworm* Infection	18 (15.4)	99 (84.6)	117 (100)	1 (0.85)	4 (3.42)	5 (4.27)	0.67	0.18
Trichuriasis	62 (73)	23 (27)	85 (100)	32 (37.6)	16 (13.7)	48 (56.5)	0.21	0.82

Abbreviation: *X* ^2^, chi‐square.

## 4 DISCUSSION

In this study, a single dose of 400 mg ALB showed, a satisfactory efficacy against *A. lumbricoides* with ERR of 97.30%, and for hookworm infections showed that, a doubtful ERR of 82.07%, and a reduced (unsatisfactory) efficacy against *T. trichiura* parasite, with ERR of 23.12%, which is much lower than the new WHO guide line.^27^ In the present study, moderate infection intensity does not affect the CR and ERR for *A. lumbricoides* and hookworm infection but for *T. trichiura*, as the infection intensity increases, the CR decreases. This may be due to *T. trichiura* infecting the lower bowel of the stomach (cecum, appendix, colon, and rectum.^28^ As a result, ALB may not be equally distributed throughout the gastrointestinal tract. Oral dosage of ALB is poorly absorbed in the gastrointestinal tract due to its limited water solubility. This could shorten the amount of time that ALB could have affected the parasite and decrease its effectiveness.

The CR of *A. lumbricoides* and Hookworm infection in this study was higher than the study conducted in seven STH‐endemic countries,^29^ but the ERR of *A. lumbricoides*, Hookworm, and Trichiura was higher in the present study. The lower CR of *A. lumbercoides* and Hookworm infection may be due to the pretreatment infection intensity (treatment history) observed in some countries like Cameroon, Tanzania, and India. The higher efficacy (ERR) occurred in the three STHs' could be attributed to the anthelmintic's high quality (intrinsic quality, bioavailability, and/or appropriate storage or transportation).^30^

In the current study, a single 400 mg ALB shown reduced CR and ERR for T. trichiura and hookworm, while a single 600 mg ALB was conducted in the villages of Kotto Barombi and Marumba II (South‐West Cameroon).^31^ showed a higher CR and ERR of 84.6% and 55.3%, respectively for *T. trichiura*, and100.0% for *N. americanus*. This may due to the dose difference in ALB. This may due to the dose difference in ALB. ALB is poorly absorbed (oral dose) in the gastrointestinal tract; as a result high dose of ALB for *T. Trichuris* and hookworm parasites really shows efficient efficacy. The overall ERR of hookworm resulting from ALB in the current study was 82.07%, above a report from riverine communities of Warri North Local Government Area of Delta State, Nigeria.^32^ Hookworm had an ERR of 64% after a single 200 mg dosage of ALB. The cause of the observed discrepancy could be due low dose of ALB and the community's pretreatment history (5% of the population had had deworming during the previous 4 years). In a study conducted in rural KwaZulu‐Natal (KZN)/South Africa,^15^ a treatment with a single dose of 400 mg ALB was very effective against hookworm and *A. lumbricoides*, with CRs of 78.8%, 96.4% and 93.2% and 97.7%, respectively. The CR and ERR for T. trichiura were 12.7% and 24.8%, respectively. The CR was low for hookworm, *A. lumbericoides*, and *T. trichuria*, but a higher ERR for hookworm and a slightly higher ERR for *T. trichuria* infection in the present study.

In this study, the CR of hookworm infection produced by a single dose of 400 mg ALB was higher than the study conducted in Lao PDR.^32^ This variation could be caused by periodic changes in hookworm egg counts from individuals, and the sample size was smaller than in the current study. The significance may be attributed to host factor and the inability of certain children to properly swallow the drug. The egg reduction rate attained in the current research for the treatment of trichuriasis (23.1%) after a single 400 mg ALB dose was lower than the ERR of 69.8% obtained in Shesha Kekel and Wondo Wosha, Southern Ethiopia,^33^ but the CR (17.1%) is lower than the present study's 43.5%. This difference may be due to the host factor, that is, the host may be affected by intestinal transit, drug absorption and bioavailability, and episodes of vomiting.^27^

In another previous study in Ethiopia (Wondo Genet, Southern Ethiopia),^28^ a single dose of 400 mg ALB (manufactured by *Khandelwal* Laboratories Pvt. Ltd.) produced a high ERR (93.3%) against hookworm, a low ERR for *A. lumbricoides* (90%), and a slightly higher ERR (25%) for *T. trichiura* than the present study. The occurrence of this difference may be due to the statistical analysis (using a geometric mean) to calculate the ERR,^27^, ^29^ that is, the arithmetic mean of egg count provides more accurate ERR results than egg counts calculated from a geometric mean, which may result in under or overestimating drug efficacy. Furthermore, a significant association “*p* < 0.001”’ swas found in the current study between age and *T. trichuris* CR. This may be due to the parasite's higher prevalence in children below 11 years; hence, the young children spend much time playing with dust matter, so they may be exposed to a high risk of infection and thus diminish their immunity.

### 4.1 Limitation of the study

This study's shortcomings included its inability to do or check drug combination for treating *T. Trichuris*. In addition, due to a lack of resources we do not perform the most sensitive and specific test like PCR (Polymerize Chain reaction) analysis and a failure to cover various drug brands and dosages. More‐over, for normally distributed data, we are unable to calculate means and standard deviations (SDs); for non‐normally distributed data we are unable to calculate medians, ranges, or interquartile ranges as well as he there is no addition of figure legend.

## 5 CONCLUSION AND RECOMMENDATIONS

This study showed that A single dose of 400 mg ALB is effective (satisfactory), doubtful, and unsatisfactory against *A. lumbricoides*, hookworm, and *T. trichiura* infections, respectively Hence, a single dose of 400 mg of ALB against hookworm and *T. trichiura* infections should be closely followed up. But when it comes to curing *T. trichiura* infections and reducing the quantity of eggs, the combination of ALB 400 mg and metendazole 500 mg as a single dose works better than ALB 400 mg alone. Further studies using different brands, doses, and routes are needed to treat the parasites of hookworm and *T. trichiura* infections successfully. Additional studies are also required to assess the efficacy of STH intervention with ALB by using a larger sample size and PCR diagnostic method.

## AUTHOR CONTRIBUTIONS

**Adane Assefa**: Conceptualization; data curation; formal analysis; investigation; methodology; project administration; resources; software; supervision; validation; visualization; writing—original draft; writing—review and editing. **Demekech Damtie**: Conceptualization; data curation; formal analysis; funding acquisition; investigation; methodology; project administration; resources; software; supervision; validation; visualization; writing—review and editing. **Getu Abeje**: Conceptualization; data curation; formal analysis; funding acquisition; investigation; methodology; project administration; resources; software; supervision; validation; visualization; writing—review and editing. **Andargachew Almaw**: Conceptualization; data curation; formal analysis; funding acquisition; investigation; methodology; project administration; resources; software; supervision; validation; visualization; writing—review and editing. **Ayenew Berhan**: Conceptualization; data curation; formal analysis; funding acquisition; investigation; methodology; project administration; resources; software; supervision; validation; visualization; writing—review and editing. **Habtu Debsh**: Conceptualization; data curation; formal analysis; funding acquisition; investigation; methodology; project administration; resources; software; supervision; validation; visualization; writing—review and editing. **Molla Getie**: Conceptualization; data curation; formal analysis; funding acquisition; investigation; methodology; project administration; resources; software; supervision; validation; visualization; writing—review and editing.

## CONFLICT OF INTEREST STATEMENT

The authors declare no conflict of interest.

## ETHICS STATEMENT

Consent for publication is not applicable as individual data such as images and videos did not accompany this manuscript.

## TRANSPARENCY STATEMENT

The lead author Getu Abeje affirms that this manuscript is an honest, accurate, and transparent account of the study being reported; that no important aspects of the study have been omitted; and that any discrepancies from the study as planned (and, if relevant, registered) have been explained.

## Supporting information

Supporting information.

## Data Availability

The original data for this study is available from the corresponding author. All authors have read and approved the final version of the manuscript had full access to all of the data in this study and takes complete responsibility for the integrity of the data and the accuracy of the data analysis.
