# Supplementary material for: Efficacy of 400 mg albendazole against soil‐transmitted helminthes among Salgy Primary School Children, Dembia district, Northwest Ethiopia, 2020. “Uncontrolled experimental study”
Source: Health Sci Rep. 2024 Apr 17;7(4):e2041. doi: 10.1002/hsr2.2041 (PMC11022293; doi:10.1002/hsr2.2041)
Supplement: Supplementary file 1 — Supporting information. [file HSR2-7-e2041-s001.docx]

**Dear academic editor**

Accompanying this letter, you will find the manuscript entitled **“”** This study was undertaken to **Efficacy of 400 mg Albendazole against Soil-transmitted helminthes among Salgy Primary School children, Dembia district, Northwest Ethiopia, 2020. “Uncontrolled experimental study”.** **An uncontrolled** experimental study was conducted at Salgy Primary School Children, Northwest Ethiopia ,from March to May 2020.

A total of 439 schoolchildren were enrolled and screened for soil-transmitted helminths by stratified proportionate systematic random sampling to get 228 positive schoolchildren. Students in grades one through eight were grouped based on their educational attainment. Using the Kato-Katz thick smear technique, the selected stool sample collected from school children was examined using the Kato-Katz thick smear technique to determine the cure and egg reduction rates. The statistical package for social science software, version 20, was used to analyze the data. To determine the relationship between CR (cure rate) and ERR (egg reduction rate) by age, a chi-square test (X 2) was employed and significance was considered at A 95% confidence interval and p-value (p < 0.05).

Therefore, A 400 mg single dosage of albendazole showed a 99.35% cure rate and a 97.30% egg reduction rate against *Ascaris lumibricoides*. Additionally, a 400 mg dose of albendazole showed a 95.75% cure rate and an 82.07% egg reduction rate, suggesting questionable effectiveness against hookworm infections. *Trichuris trichiura* showed a decreased efficacy, with a 43.53% cure rate and a 23.12% egg reduction rate.The manuscript is submitted only to your prestigious journal. All authors agreed and approved the manuscript. Authors have no any conflict of interest. We are looking forward to your consideration of this manuscript in your prestigious journal.

Best regards,

Getu Abeje (MSc in Medical Parasitology and vector control)

**Email:** [**getuabeje2121@gmil.com**](mailto:getuabeje2121@gmil.com)

Cell phone: +251979508585/982526731

Samarar University,Samara ,Ethiopia
